# Painful Menstruation in Virgins

**Published:** 1890-08

**Authors:** I. N. Love


### PAINFUL MENSTRUATION IN VIRGINS.

BY DR. I. N. LOVE.

Some workers in the field of diseases of women believe that the ills to which female flesh is heir can properly be charged to the reproductive organs, but the majority of broad-gauge practical physicians know that the "better-half" of mankind are liable to all forms of lesion and suffering which may be charged to all the individual portions of the animal economy.

The *Golden Gems* of *Goodell*, recently enunciated, should be printed in large type and placed prominently before the eyes of every doctor in the sacred sanctum wherein he mostly pursues his studies. They are as follows, viz :

First, always bear in mind that "women have organs outside of the pelvis."

Second, each neurotic case will usually have a tale of fret or grief, of cark and care, of wear and tear.

Third, scant or delayed, or suppressed menstruation is far more frequently the result of nervous exhaustion than of uterine disease.

Fourth, anteflexion is not *per se* a pathological condition. It is so when associated with sterility or painful menstruation, and only then does it need treatment.

Fifth, an irritable bladder is more often a nerve symptom than a uterine one.

Sixth, in a large number of cases of supposed or of actual uterine disease which displays marked gastric disturbance, if the tongue be clean, the essential disease will be found to be neurotic, and must be treated as such.

Seventh, almost every supposed uterine case, characterized by excess of sensibility and by scantiness of will power, is essentially a neurosis.

Eighth, in the vast majority of cases in which a woman takes to bed and stays there indefinitely, from some supposed uterine lesion, she is bedridden from her brain and not from her womb.

Lastly, uterine symptoms are not always present in cases of uterine disease, nor when present, even urgent, do they necessarily come from uterine disease, for they may be merely nerve counterfeits of uterine disease.

These are the cases of painful or difficult menstruation particularly in virgins which seriously burden the conscientious physician. Many such have come under my observation, and I have always felt that every general therapeutic means should be exhausted before local measures should be instituted; in fact in nearly all cases special treatment should be avoided with young girls.

For about six months I have treated numerous cases of such character with *Ponca Compound* in tablet form; actuated thereto by the suggestions thrown out by Dr. Deering J. Roberts, of Nashville, and Dr. W. T. Dixon, of Evansville, Ind. The Ponca Compound being presented to the profession by the Mellier Drug Co., of St. Louis, a name which has been synonymous with honesty, reliability and skill in pharmacy in St. Louis for almost half a century, I did not hesitate to use it. I find that each tablet contains Ex. Ponca, 3 grs.; Ext.

Mitchella Repens, 1 gr.; Caulophyllin, 1-4 gr.; Helonin 1-8 gr.; Viburnin, 1-8 gr.

I usually administer one tablet every four hours, and so far am much gratified with the results. I feel that any remedy which will help us out in these cases should be welcomed.—*Medical Mirror.*

